# A mobile health intervention for improving the technique of inhaled medications among children with asthma: A pilot study

**DOI:** 10.1177/20552076231216589

**Published:** 2023-11-28

**Authors:** Mary Jane Smith, Zhiwei Gao, Roger Chafe, Meshari Alwashmi

**Affiliations:** 1 Faculty of Medicine, 7512 Memorial University of Newfoundland, St John’s, Canada; 2 Janeway Children’s Health and Rehabilitation Centre, Eastern Health, St John’s, Canada; 3 BreatheSuite, St John’s, Canada

**Keywords:** Mobile health, asthma, pediatric care, intervention, patient satisfaction, Canada

## Abstract

**Objective:**

BreatheSuite MDI is a Bluetooth-enabled inhaler attachment and mobile application which aims to improve asthma control. The objective was to compare pressurized metered dose inhaler (pMDI) technique and asthma control test (ACT) scores pre- and post-use of the device and mobile application. Secondary objectives were to assess user satisfaction and therapy adherence.

**Methods:**

Patients between the ages of 8 and 18 were recruited from several pediatric asthma clinics. Technique and ACT scores were assessed at baseline. Users were given no prompts on technique during the first month of device use. For the subsequent three months, users were given technique scores through the mobile application after each inhaler use and provided weekly performance summaries. At the end of the study, technique and ACT scores were analyzed and an exit survey was completed. Conditional logistic regression was used to examine the association between well-controlled asthma (ACT score > 19) and the intervention.

**Results:**

24 patients completed the study. Technique scores improved following the use of Breathesuite (44.19 vs. 62.54; *P* = 0.01). Well-controlled asthma did not significantly improve (OR = 1.20 [0.4–3.9], *P* = 0.76). 87% of study subjects agreed or strongly agreed that their asthma control improved while using BreatheSuite; 79% were satisfied with the device and mobile application; and 91% preferred using the device compared to a standard logbook to track inhaler usage.

**Conclusions:**

In this pilot study, the use of BreatheSuite device was associated with improved technique scores. These results need to be confirmed by a randomized controlled trial. There was high user satisfaction with the BreatheSuite device.

## Introduction

Asthma is one of the most common chronic diseases in childhood. In Canada, it is the most common cause for hospitalization within the pediatric population^1^ and has a lifetime prevalence between 11% and 16%.^2^ Between 3% and 7% of all emergency room visits among children are for asthma exacerbations.^3^ Asthma symptoms are also a leading reason for school absences.^4^

Asthma in children can be diagnosed clinically, and depending on age, in conjunction with pulmonary function testing.^5,6^ Once the diagnosis is confirmed, treatment for all ages usually includes patient education, environmental control and pharmacotherapy. Many asthma medications are delivered by inhaler devices. These devices come in many forms, including pressured metered dose inhalers (pMDI). Using valved holding chambers (VHC) with pMDI can help overcome poor hand-mouth coordination and reduce side effects, with increased drug delivery and lung deposition.^5^

While inhalers can be an effective method for delivering asthma medications, traditional inhalers do have some drawbacks. Studies have reported that up to 92% of patients with asthma demonstrate poor inhaler technique.^7–9^ Press et al. found regular inhaler misuse among patients with asthma.^10^ Improper inhaler technique can significantly affect the amount of medication reaching the lungs, leading to poor symptom control and more emergency department visits.^11,12^ Errors in inhaler technique and nonadherence can affect medication delivery and decrease the benefits of taking the medication.^13^ A systematic review of errors in inhaler technique suggests that most reported errors were in coordination, speed of the inhalation, depth of inspiration and no post-inhalation breath-holding.^14^ To improve performance, researchers recommend frequent assessment of inhalation techniques.^15,16^ There is a gap in the current academic literature about what is the most appropriate intervention if patients continue to misuse their inhalers.^16^

Innovative technologies have been introduced which may improve inhaler technique and consequently improve health outcomes.^17–19^ There have been significant advances in digital health interventions, including the development of mobile apps, web portals, and electronic inhaler sensors. Digital health applications now exist for many complex health conditions, including asthma. These technologies allow patients and health care providers to monitor and manage their symptoms more effectively. Several studies demonstrated improved clinical outcomes from implementing these technologies.^17,19–21^ A recent meta-analysis determined that electronic reminders can improve patient adherence to inhaled corticosteroids by 19%.^22^ Another advantage of using digital health applications related to asthma is having long-term data collection of symptoms, triggers and inhaler use. This data can identify periods of exacerbating symptoms or the need to change a patient's care plan.^23,24^ Advancements in electronic medical devices could further enhance asthma management.

The BreatheSuite MDI device is an auxiliary, add-on device which is connected to an approved pMDI inhaler. It passively monitors important inhaler adherence and technique metrics, providing the patient feedback through a linked mobile app. This information may be used by patients to improve inhaler technique and can be shared with healthcare providers. There are several devices that monitor the adherence of pMDIs,^25^ but few have the capacity to monitor both adherence and technique. The BreatheSuite MDI can detect both.^26^ This study compares patients’ pMDI technique and asthma control test (ACT) scores pre and post-use of BreatheSuite. Secondary objectives were to assess user satisfaction and therapy adherence using the device.

## Methods

### Breathesuite MDI

**Figure 1.**
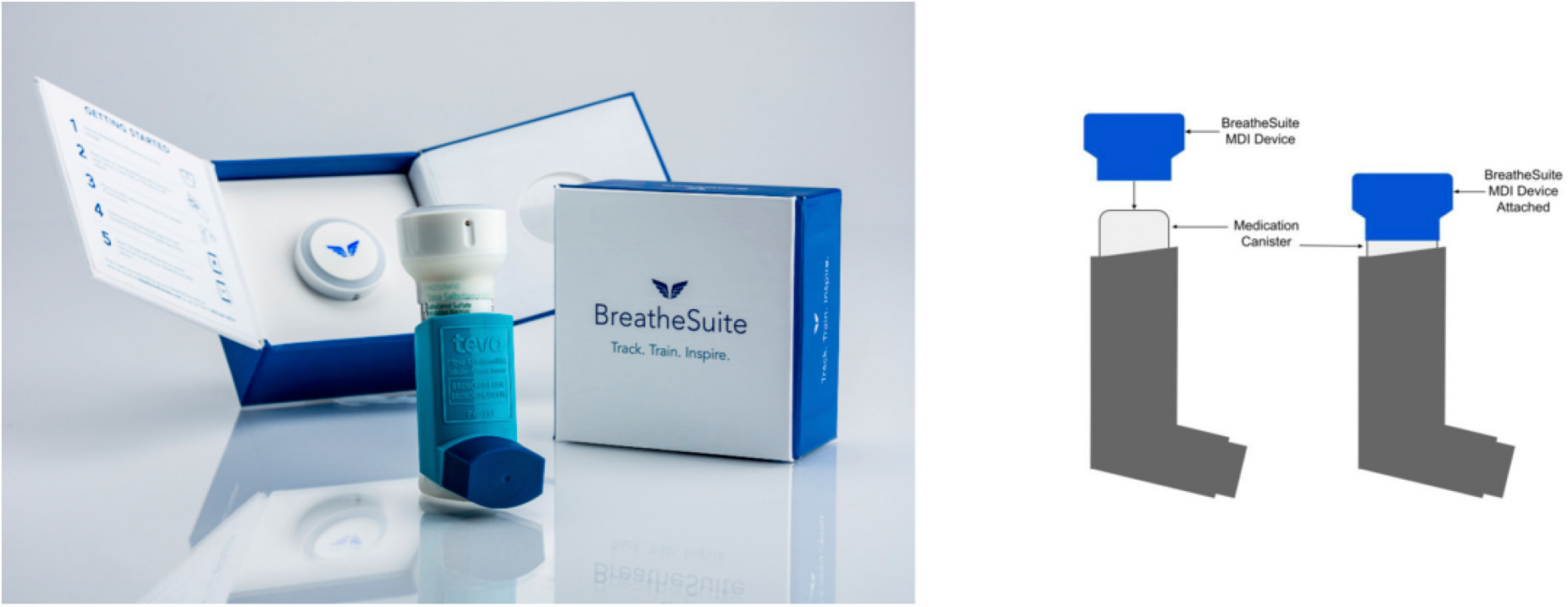
Placebo inhaler with the BreatheSuite metered-dose inhaler device.

The BreatheSuite MDI device is approximately 1 inch in diameter and attaches to a standard MDI canister with an elastic sleeve (Figure 1). The device is designed for passive monitoring, with the operation of the inhaler not being affected by its installation.

The device assesses several inhaler technique metrics. The metrics monitored were selected based on inhaler manufacturers’ instructions, as well as quantitative analysis performed on lung deposition related to inhaler technique parameters. Specifically, the BreatheSuite MDI device captures data on the following:

1. Shaking duration: The goal is for patients to shake their inhaler for at least 3 s before use.
2. Orientation: The goal is for the inhaler to be oriented with the mouthpiece straight toward the back of the throat.
3. Press timing: The goal is for the user not to begin inhaling before actuating the inhaler canister.
4. Inhalation duration: The goal is for the user to inhale for at least 3 s.

It is important to note that all participants used a VHC and only two technique metrics were consistently tracked (shaking and orientation) during the study. This information is transmitted to the BreatheSuite mobile app via Bluetooth. During the study period, the user received technique-related feedback and subsequent correcting advice by accessing the mobile app. The data is transferred from the app to a secure database to be available for further analysis and review by a healthcare provider.

### Recruitment and study setting

A convenience sample of 30 study subjects was recruited. A research nurse invited patients to participate if they met all of the following eligibility criteria: were between 8 and 18 years of age; had a pre-existing diagnosis of asthma confirmed by a pediatrician; had regular access to a smartphone; could demonstrate proper technique of pMDI with VHC while supervised by an asthma nurse or pediatrician without parent or caregiver intervention; and had an ongoing need for an asthma medication delivered via pMDI as deemed by a pediatrician. Medications included short-acting beta-agonists, inhaled corticosteroids and inhaled corticosteroid/long-acting beta-agonists. Study subjects were recruited from asthma clinics at the Janeway Children's Health and Rehabilitation Centre (St John's, Canada). Recruitment occurred either in person or virtually during scheduled appointments with pediatricians.

### Study design and data collection

The study was a prospective interventional pilot study. Baseline pMDI technique was assessed according to the Canadian Thoracic Society Asthma Guidelines.^27^ Participants were coached about the proper inhaler technique and completed the ACT. The ACT is a brief, validated patient-reported assessment of asthma symptoms and impact that evaluates the impairment domain of asthma control in patients with asthma.^28^ The BreatheSuite devices were given or mailed to subjects and they were instructed on how to use it by an asthma nurse via Microsoft Teams. All subjects were given Asthma Action Plans, which detailed how frequently and in what circumstances medications should be used. In addition, they were instructed to record their doses manually in a provided paper-based asthma logbook.

The first 30 days of the study were designated as the “baseline phase,” during which asthma control and inhaler use were assessed. During this period, inhaler usage and technique data were monitored, but neither the participants nor the clinicians had access to the data. After the 30-day baseline phase, participants were provided with a weekly summary and tips on how to improve their technique via email based on the data collected for that participant. This phase of the study lasted 3 months after the baseline period.

Four months after recruitment, participants completed an assessment with the research nurse. They were requested to demonstrate proper MDI technique and completed a second ACT. Available logbooks were collected. In addition, data from the BreatheSuite MDI was downloaded and reviewed. All subjects completed exit surveys that included questions about their experiences and their perceptions of their asthma control. Surveys were developed by the research team based on a review of similar evaluation questionnaires and to meet the study objective.

ACT was scored as per the defined criteria.^29^ Children with ACT score of > 19 were considered to have well-controlled asthma. Medication usage was considered adherence when medication usage equaled expected usage. ACT was used off label in children under 12 with parental input into the answers.

### Statistical analysis

Descriptive statistics were calculated for continuous and categorical variables respectively. Conditional logistic regression was used to examine the association between well-controlled asthma (ACT score > 19) and the use of the intervention. Paired t-tests were used to examine the differences in the average percentage of adherence and average technique score between the baseline and follow-up periods. Statistical analysis was carried out by SAS 9.4®.

## Results

We identified 30 participants who met our study inclusion criteria. Four potential subjects did not complete a consent form or failed to respond to contact attempts. Of the 26 study subjects who began, two did not complete the study. We were not able to follow-up with one study subject after study initiation. The other discontinued the study due to perceived increase in symptoms when their VHC technique was corrected prior to study initiation. 24 participants completed the entire study, consisting of 14 (58%) males and 10 females (42%), with a mean age of 11.4 years old (Table 1).

**Table 1.** Study population and intervention results.

Study population	
Sex, N (%)	
Males	14 (58)
Females	10 (42)
Age (years), Mean (STD)	11.4 (2.47)
Intervention	
Well-controlled asthma, N (%)	
Baseline	17 (70.8%)
Post-intervention	18 (75.0%)
Odds ratio (reference: Baseline) (95% CL; P value)	1.2 (0.4, 3.9); P = 0.7633
Average technique score, Mean (STD)	
Baseline	44.2 (20.43)
Post-intervention	62.5 (20.98)
Difference in means (95% CL; P value)	18.3 (9.1, 27.6); P = 0.0005
Adherence, mean	
Baseline	47.00
1 month post-intervention	46.70
2 month post-intervention	37.10
3 month post-intervention	7.89

There was an increase in the average technique score between the baseline and intervention periods (44.2 vs. 62.5; P = 0.0005). There was also a slight non-significant increase in the number of participants with Well-Controlled Asthma during the intervention period (70.8% vs. 75%; P = 0.7633). Adherence scores remained fairly consistent during the baseline period and the first month of the intervention (47.00% vs. 46.70%). They fell during the second and third months of the intervention.

Responses to the post-intervention survey were positive (Table 2). 95.7% found that the device helped improve their technique. 87% felt their asthma control improved while using the device. Among study subjects, 91.3% preferred tracking their adherence via the BreatheSuite MDI application over the paper logbook. In response to open-ended survey questions, participants agreed that the device has the potential to enhance inhaler technique. One patient remarked: “My inhaler technique improved because of the written feedback I received from the device. It improved the quality of my dose and the adherence to my dosing.” Another patient described that the device could provide a sense of motivation: “The device encouraged me to use my inhaler more as I knew I was being recorded.” Some participants had concerns about connecting their device to the smartphone: “I had issues with the device connecting to the App in the beginning of the study… The device ended up being replaced. I had no issues with the new device.”

**Table 2.** Results of post-intervention surveys.

Question	Strongly agreed or agreed, N (%)
I was overall satisfied with the device and mobile application/“app”.	18 (78.3)
I was satisfied with the information sent to me after each dose and in the weekly emails.	17 (73.9)
This device helped me improve my inhaler technique.	22 (95.7)
This device and mobile application were easy to use.	18 (78.3)
This device was easy to carry around.	20 (87.0)
I would be interested in continuing to track my asthma medication usage with this program.	17 (73.9)
I prefer the device and mobile application over the logbook to track my inhaler use.	21 (91.3)
I missed fewer doses when using the device and application compared to the logbook alone.	16 (69.6)
I feel my asthma control improved while using the device and mobile application.	20 (87.0)

## Discussion

This study was the first to assess a Bluetooth-enabled inhaler's ability to measure adherence and technique within a pediatric population with asthma. The study showed a significant improvement in technique scores following the use of the BreatheSuite MDI device. The inhaler was able to successfully report adherence data. There was a small increase in cases of well-controlled asthma reported. There was also a high level of patient satisfaction with the device and the mobile application, with most participants reporting that the device helped with their asthma control and was preferable to traditional methods for tracking medication usage.

The increase in participants’ technique scores is encouraging. Asthma is one of the leading causes of hospitalization and emergency department usage in the pediatric population. Improvements in the way patients self-administer their medication would address one potential factor leading patients to seek care. The device also demonstrated the ability to capture and report adherence data. Adherence was measured by comparing inhaler usage with expected medication usage. The percentage of participants adhering to expected usage declined over the study period. There are several possible reasons for this decline, including that the devices were not replaced after patients received new inhaler refills or that there were issues with the battery, issues that have been addressed in later models of the device. Because few participants returned their logbooks, we cannot determine whether doses were in fact administered or if the issue was with the data reported by the device. If indeed there was a drop off in usage, it may suggest that an intervention may be needed after 2 months of follow-up to prevent a drop in medication adherence.

Well-controlled asthma was less impacted by the BreatheSuite MDI. While inhaler technique is one-factor impacting asthma control, there are other relevant factors. There are several Bluetooth-enabled attachments for asthma inhalers currently in development. The ability to impact asthma exasperation and ultimately health services usage will require that the right mixture of monitoring and reporting capabilities are built into these devices. This study adds to the critical evidence base about the potential capabilities of inhalers connected to mobile applications.

This study had several limitations. The patient population all came from a convenience sample from one Canadian province. Most study participants had well-controlled asthma throughout the study and were able to consistently adhere to study guidelines for a 4-month period. In future studies, we plan to recruit more participants who have uncontrolled asthma. Including those who have uncontrolled asthma will likely allow us to better assess the impact of using a Bluetooth-enabled inhaler on asthma control and rescue medication usage. We also plan to explore the effect of patient-directed mobile applications combined with expanded digital coaching on asthma management and healthcare utilization.

## Conclusion

Bluetooth-enabled attachments for asthma inhalers with related mobile applications will likely become the standard of patient care sometime in the near future. Making sure that these devices are effective and include the most appropriate capabilities for supporting asthma patients is crucial. While there is clearly promise in these devices and mobile applications, more work needs to be done to ensure we are maximizing their capabilities for patients and providers. In this study, we found that the BreatheSuite MDI was associated with the potential to enhance the MDI technique among children with asthma. In addition, most users were satisfied with the intervention. This evidence further supports both the viability of Bluetooth enabled inhalers and the ability of the BreatheSuite MDI to effectively measure both technique and medication adherence while being acceptable to most patients.
